# *Streptococcus pneumoniae* colonisation in children and adolescents with asthma: impact of the heptavalent pneumococcal conjugate vaccine and evaluation of potential effect of thirteen-valent pneumococcal conjugate vaccine

**DOI:** 10.1186/s12879-016-1335-3

**Published:** 2016-01-12

**Authors:** Susanna Esposito, Leonardo Terranova, Maria Francesca Patria, Gian Luigi Marseglia, Michele Miraglia del Giudice, Alessandro Bodini, Alberto Martelli, Eugenio Baraldi, Oscar Mazzina, Claudia Tagliabue, Amelia Licari, Valentina Ierardi, Mara Lelii, Nicola Principi

**Affiliations:** 1 Pediatric Highly Intensive Care Unit, Department of Pathophysiology and Transplantation, Università degli Studi di Milano, Fondazione IRCCS Ca’ Granda Ospedale Maggiore Policlinico, Via Commenda 9, Milan, 20122 Italy; 2 Pediatric Clinic, University of Pavia, IRCCS Policlinico “S. Matteo” Foundation, Pavia, Italy; 3 Pediatric Clinic, II Naples University, Naples, Italy; 4 Pediatric Clinic, University of Verona, Verona, Italy; 5 Pediatric Unit, Garbagnate Hospital, Garbagnate, Italy; 6 Pediatric Clinic, University of Padua, Padua, Italy; 7 Allergology Unit, IRCCS Bambino Gesù Hospital, Rome, Italy

**Keywords:** Asthma, Asthmatic children, Chronic respiratory disease, PCV7, PCV13, Pneumococcal colonisation, Pneumococcal conjugate vaccine, Pulmonary disease, *Streptococcus pneumoniae*

## Abstract

**Background:**

The main aim of this study was to evaluate *Streptococcus pneumoniae* carriage in a group of school-aged children and adolescents with asthma because these results might indicate the theoretical risk of invasive pneumococcal disease (IPD) of such patients and the potential protective efficacy of the 13-valent pneumococcal conjugate vaccine (PCV13).

**Methods:**

Oropharyngeal samples were obtained from 423 children with documented asthma (300 males, 70.9 %), and tested for the autolysin-A-encoding (*lytA*) and the *wzg* (*cpsA*) gene of *S. pneumoniae* by means of real-time polymerase chain reaction.

**Results:**

*S. pneumoniae* was identified in the swabs of 192 subjects (45.4 %): 48.4 % of whom were aged <10 years, 46.9 % aged 10–14 years, and 4.7 % aged ≥15 years (p < 0.001). Carriage was significantly less frequent among the children who had received recent antibiotic therapy (odds ratio [OR 0.41]; 95 % confidence interval [95 % CI] 0.22–0.76). Multivariate analyses showed no association between carriage and vaccination status, with ORs of 1.05 (95 % CI 0.70–1.58) for carriers of any pneumococcal serotype, 1.08 (95 % CI 0.72–1.62) for carriers of any of the serotypes included in 7-valent pneumococcal conjugate vaccine (PCV7), and 0.76 (95 % CI 0.45–1.28) for carriers of any of the six additional serotypes of PCV13. Serotypes 19 F, 4 and 9 V were the most frequently identified serotypes in vaccinated subjects.

**Conclusions:**

These results showed that carriage of *S. pneumoniae* is relatively common in all school-aged children and adolescents with asthma, regardless of the severity of disease and the administration of PCV7 in the first years of life. This highlights the problem of the duration of the protection against colonisation provided by pneumococcal conjugate vaccine, and the importance of re-colonization by the same pneumococcal serotypes included in the previously used vaccine.

## Background

School-aged children and adolescents with asthma were long thought to be not at any increased risk of invasive pneumococcal disease (IPD), and were excluded from the subjects considered for pneumococcal vaccine administration [1]. However, some recent studies designed to evaluate the possible relationship between asthma and the incidence of IPD have highlighted the importance of asthma in increasing the risk of pediatric IPD [2], and official recommendations were modified to include children aged 6–18 years (at least those treated with high-dose corticosteroids) among the patients with chronic underlying disease for whom pneumococcal vaccine should be strongly recommended [3].

The most recent recommendations indicate that school-aged children and adolescents with asthma should now receive the 23-valent polysaccharide vaccine (PPSV23) rather than the 13-valent conjugate vaccine (PCV13) [3]. This decision may be questioned because, although it contains fewer pneumococcal serotypes than PPSV23, PCV13 may offer greater protection as it is more immunogenic [4] and has a significantly greater effect on pharyngeal carriage [5]. However, there are no published data concerning PCV13 potential impact on the incidence of IPD in asthmatic children.

Carriage is a prerequisite for the development of IPD [6], and the different conjugate vaccines reduce the incidence of IPD in vaccinated subjects mainly by affecting nasopharyngeal colonisation with the pneumococcal serotypes they contain [7]. Moreover, the reduced circulation of these serotypes has positive effects also in unvaccinated subjects who are significantly less colonized than in the period before vaccine use [7]. As evaluating pneumococcal colonisation is considered a possible means of measuring the efficacy of conjugate vaccine [8], the characteristics of carriage before vaccination may offer information regarding its potential coverage and real efficacy.

The main aim of this study was to evaluate *Streptococcus pneumoniae* carriage in a group of school-aged children and adolescents with asthma because these results might indicate the theoretical risk of IPD of such patients and the potential protective efficacy of PCV13. Moreover, although it was licensed in Italy in 2000, the heptavalent conjugate vaccine (PCV7) was not widely used until 2009 even in children with chronic severe underlying disease [9], and so evaluating children born before this year may make it possible to compare carriage in both vaccinated and unvaccinated asthmatic pediatric patients and thus measure the long-term impact of PCV7.

## Methods

### Patient enrolment and swab collection

The protocol was approved by the Ethics Committees of the seven hospitals (in Milan, Ethics Committee of Milan Area B Fondazione IRCCS Ca’ Granda Ospedale Maggiore Policlinico; in Pavia, Ethics Committee of IRCCS Policlinico “S. Matteo” Foundation; in Garbagnate, Ethics Commiitee of Milan Area C Niguarda Hospital; in Padua, Ethics Committee of Padua Hospital; in Verona, Ethics Committee of Verona province; in Rome, Ethics Committee of IRCCS Bambino Gesù Hospital; in Naples, Ethics Committee of Policlinico Hospital) whose Pediatric Units enrolled asthmatic patients aged 6–17 years, regularly attending their Respiratory Disease Centres between January 2014 and December 2014 after written consent from both parents had been collected and written assent from children if aged ≥8 years had been obtained. The characteristics of the asthma were evaluated on the basis of the Global Initiative for Asthma criteria [10]. In all of the patients, allergic sensitization was previously determined by the evaluation of total IgE levels and IgE antibodies to food allergens (cow’s milk, egg white, and wheat), indoor allergens (house dust mite and cat dander), and outdoor allergens (mixed grass and birch pollen) by means of CAP-RAST FEIA (Pharmacia, Freiburg, Germany). All of the patients were clinically stable at the time of enrolment; patients with active respiratory infection, those who had received antibiotic therapy during the previous 2 weeks, and those with a chronic underlying disease other than asthma were excluded. After enrolment, the clinical and laboratory data of each child for the previous 3 months were retrieved from their clinical records and recorded in an electronic database specifically prepared for the study.

The patients’ pneumococcal vaccination status was established by consulting the official vaccination chart issued by the Vaccination Service of the Region in which they lived. The pneumococcal immunisation schedule recommended by the Italian Ministry of Health involves three doses of PCV7 in the first year of life, or two in the second year, or a single dose after the second year until the fifth [11]. The patients were considered fully vaccinated if one of these recommendations had been fulfilled by the time of enrolment, and not fully vaccinated if they had started but not completed the vaccine schedule: as the latter group consisted of only 1 % of the enrolled subjects it was not compared with the groups of fully vaccinated or unvaccinated children.

The oropharyngeal samples were obtained using an ESwab kit containing a polypropylene screw-capped tube filled with 1 mL of liquid Amies medium (Brescia, Copan, Italy). The sampling was carried out by pressing the tongue downward to the floor of the mouth with a spatula, and swabbing both tonsillar arches and the posterior nasopharynx without touching the sides of the mouth. All of the swabs were immediately frozen at −20 °C, and transported within a week to the central laboratory where they were processed within 2 h of arrival.

### Identification of *S. pneumoniae*

Bacterial genomic DNA was extracted from the samples using a NucliSENS easyMAG automated extraction system (BioMeriéux, Bagno a Ripoli, Florence, Italy), a 250 μL sample input and a generic protocol, and was tested for the autolysin-A-encoding (*lytA*) and the *wzg* (*cpsA*) gene of *S. pneumoniae* by means of real-time polymerase chain reaction (PCR) as previously described [12]. Level of detection of the test was 16 genome copies, and each sample was tested in triplicate and considered positive if at least two of the three tests revealed the presence of both genes. In order to maximise sensitivity, no internal amplification control was used in the reaction, but there was an external control. The real-time PCR-negative specimens were also tested for the presence of an RNase P-encoding gene in order to exclude PCR inhibition and DNA extraction failure.

All of the positive cases were serotyped using primers and probes designed on the basis of the GenBank database sequences (www.ncbi.nlm.nih.gov) of serotypes 1, 3, 4, 5, 6A, 6B, 7 F, 9 V, 14, 18C, 19A, 19 F and 23 F (i.e. those in the 13-valent pneumococcal conjugate vaccine, PCV13), and synthesised by TIB Molbiol (Genoa, Italy) as previously described [12]. Analytical specificity was pre-evaluated by means of computer-aided analyses using Primer-blast (www.ncbi.nlm.nih.gov/tools/primer-blast) and blast software (www.blast.ncbi.nlm.nih.gov/Blast.cgi) in order to compare the sequences with all of the sequences listed under ‘bacteria’ and ‘homo sapiens’.

### Statistical analysis

The groups were compared using the *χ* ^2^ or Fisher’s exact test as appropriate. The ordered categorical data were compared using a Cochran-Armitage test for trend. Multivariate odds ratios (ORs) and 95 % confidence intervals (CIs) were calculated using unconditional multiple logistic regression models in order to measure the associations between: i) pneumococcal vaccination and pneumococcal carrier status; and ii) selected demographic and clinical characteristics and pneumococcal carrier status. Adjustments were made for the covariates defined *a priori*: i.e., age (i.e., 6–9 years, 10–14 years, ≥15 years), gender, ethnicity, the number of siblings, and parental smoking habits. Stratified analyses were also used for the two major age subgroups (<10 and 10–14 years). All of the analyses were two tailed, and *p*-values of 0.05 or less were considered statistically significant. All of the analyses were made using SAS version 9.2 (Cary, NC, USA).

## Results

Table 1 shows the characteristics of the 423 enrolled children with documented asthma (300 males, 70.9 %; mean age ± standard deviation, 10.8 ± 2.8 years): 176 (41.6 %) were aged <10 years; 207 (48.9 %) aged 10–14 years, and 40 (9.5 %) aged ≥15 years. *S. pneumoniae* was identified in the swabs of 192 subjects (45.4 %). Carriage was more frequent in the younger subjects and declined with age (48.4 % aged <10 years, 46.9 % aged 10–14 years and 4.7 % aged ≥15 years; p < 0.001), and significantly more frequent among children born at term than in those born pre-term (*p* = 0.005). There were no differences between carriers and non-carriers in terms of gender, ethnicity, the number of siblings, parental smoking habits, birth weight, exclusive breast feeding, allergic sensitisation, or meningococcal or influenza vaccination.

**Table 1:** Main characteristics of 423 children and adolescents with asthma, by pneumococcal carriage

Characteristic	All children (*n* = 423) n (%)	Carriers (*n* = 192) n (%)	Non-carriers (*n* = 231) n (%)	*p*-value
Age				
<10	176 (41.6)	93 (48.4)	83 (35.9)	
10–14	207 (48.9)	90 (46.9)	117 (50.6)	
≥15	40 (9.5)	9 (4.7)	31 (13.4)	**<0.001**
Gender				
Male	300 (70.9)	136 (70.8)	164 (71.0)	
Female	123 (29.1)	56 (29.2)	67 (29.0)	0.97
Ethnicity				
Caucasian	372 (87.9)	170 (88.5)	202 (87.5)	
Non-Caucasian	51 (12.1)	22 (11.5)	29 (12.5)	0.73
No. of siblings^a^				
0	100 (23.8)	46 (24.2)	54 (23.5)	
1	217 (51.7)	99 (52.1)	118 (52.3)	
2	82 (19.5)	37 (19.5)	45 (19.6)	
≥3	21 (5.0)	8 (4.2)	13 (5.6)	0.63
Parental smoking habit				
Both non-smokers	276 (65.2)	119 (62.0)	157 (68.0)	
At least one smoker	147 (34.8)	73 (38.0)	74 (32.0)	0.20
Gestational age (weeks)^a^				
<37	49 (11.7)	13 (6.8)	36 (15.7)	
≥37	370 (88.3)	177 (93.2)	193 (84.3)	**0.005**
Birth weight (g)^a^				
<2500	39 (9.3)	13 (6.8)	26 (11.5)	
≥2500	378 (90.7)	177 (93.2)	201 (88.5)	0.11
Exclusive breastfeeding^a^				
No	139 (32.9)	56 (29.3)	83 (35.9)	
Yes	283 (67.1)	135 (70.7)	148 (64.1)	0.15
Allergic sensitisation				
No	59 (14.0)	26 (13.5)	33 (14.3)	
Yes	364 (86.0)	166 (86.5)	198 (85.7)	0.83
Meningococcal vaccination				
No	227 (53.7)	99 (51.6)	128 (55.4)	
Yes	196 (46.3)	93 (48.4)	103 (44.6)	0.43
Flu vaccination during current season^a^				
No	351 (83.0)	162 (84.4)	189 (81.8)	
Yes	72 (17.0)	30 (15.6)	42 (18.2)	0.49

^a^Some missing values

Bold data represent significant differences

Table 2 shows the associations between demographic and clinical characteristics and pneumococcal carriage. Carriage was not associated with parental smoking habits (OR 1.36; 95 % CI, 0.90–2.07), systemic corticosteroid therapy in the previous 3 months (OR 1.03; 95 % CI 0.59–1.81), the degree of severity of asthma (OR 1.02, 95 % CI 0.65–1.61 for moderate persistent asthma and OR 0.90, 95 % CI 0.35–2.27 for severe persistent asthma), or the occurrence of respiratory relapses in the previous 3 months (OR 0.70; 95 % CI 0.45–1.10), but was significantly less frequent among the children who had received recent antibiotic therapy (OR 0.41; 95 % CI 0.22–0.76).

**Table 2:** Association between demographic and clinical characteristics and pneumococcal carriage in children with asthma

	Carriers (*n* = 192)	Non-carriers (*n* = 231)	Crude OR (95 % CI)	OR (95 % CI)^a^
	n (%)	n (%)		
Age				
<10	93 (48.4)	83 (35.9)	1 (reference)	1 (reference)
10–14	90 (46.9)	117 (50.6)	0.69 (0.46–1.03)	0.69 (0.46–1.04)
≥15	9 (4.7)	31 (13.4)	0.26 (0.12–0.58)	0.25 (0.11–0.56)
Gender				
Male	136 (70.8)	164 (71.0)	1 (reference)	1 (reference)
Female	56 (29.2)	67 (29.0)	1.01 (0.66–1.54)	0.94 (0.61–1.45)
Siblings^b^				
No	46 (24.2)	54 (23.5)	1 (reference)	1 (reference)
Yes	144 (75.8)	176 (76.5)	0.96 (0.61–1.51)	0.96 (0.61–1.53)
Parental smoking habit				
Both non-smokers	119 (62.0)	157 (68.0)	1 (reference)	1 (reference)
At least one smoker	73 (38.0)	74 (32.0)	1.30 (0.87–1.95)	1.36 (0.90–2.07)
Asthma type				
Intermittent	40 (20.8)	34 (14.7)	1 (reference)	1 (reference)
Mild, persistent	64 (33.3)	80 (34.6)	0.68 (0.39–1.19)	0.66 (0.37–1.17)
Moderate, persistent	79 (41.1)	103 (44.6)	0.65 (0.38–1.12)	0.67 (0.38–1.18)
Severe, persistent	9 (4.7)	14 (6.1)	0.55 (0.21–1.42)	0.59 (0.22–1.57)
Systemic corticesteroid therapy (previous 3 mos)^b^				
No	158 (84.0)	194 (85.8)	1 (reference)	1 (reference)
Yes	30 (16.0)	32 (14.2)	1.15 (0.67–1.98)	1.03 (0.59–1.81)
Antibiotic therapy (previous 3 months)^b^				
No	171 (90.5)	184 (81.8)	1 (reference)	1 (reference)
Yes	18 (9.5)	41 (18.2)	0.47 (0.26–0.85)	0.41 (0.22–0.76)
Respiratory relapses (previous 3 months)^b^				
No	144 (76.2)	159 (70.3)	1 (reference)	1 (reference)
Yes	45 (23.8)	67 (29.7)	0.74 (0.48–1.15)	0.70 (0.45–1.10)
Asthma under control^b^				
No	33 (17.5)	54 (23.9)	1 (reference)	1 (reference)
Yes	156 (82.5)	172 (76.1)	1.48 (0.91–2.41)	1.41 (0.86–2.31)

^a^Multivariate model adjusted for age, gender, number of siblings, and parental smoking

^b^Some missing values. 95 % CI: 95 % confidence interval; OR: odds ratio

Table 3 shows the relationship between pneumococcal vaccination status and pneumococcal carriage in the population as a whole and the two younger age groups (there were too few vaccinated children in the group aged ≥15 years). The proportions of carriers of any pneumococcal serotype and any serotype included in PCV7 were higher among the vaccinated subjects (47.7 vs. 43.4 % among non-vaccinated subjects for any serotype, and 45.1 vs. 39.9 %, respectively, for any serotype included in PCV7), whereas the proportion of carriers of the six additional serotypes included in PCV13 were higher among the unvaccinated children (18.9 %, as compared to 16.9 % among vaccinated subjects). However, multivariate analyses showed that there was no association between carriage and vaccination status: OR 1.05 (95 % CI 0.70–1.58) for carriers of any pneumococcal serotype, 1.08 (95 % CI 0.72–1.62) for carriers of any serotype in PCV7, and 0.76 (95 % CI 0.45–1.28) for carriers of any of the six additional serotypes in PCV13. The age group sub-analysis showed similar results without any differences between vaccinated and unvaccinated subjects.

**Table 3:** Relationship between pneumococcal vaccination status and pneumococcal carriage in children with asthma

	Vaccinated with PCV7 (*n* = 195) n (%)	Not vaccinated against pneumococcus (*n* = 228) n (%)	Crude OR (95 % CI)	OR (95 % CI)^a^
Pneumococcal carrier status				
Any serotype				
Non-carriers	102 (44.2)	129 (55.8)	1 (reference)	1 (reference)
Carriers	93 (48.4)	99 (51.6)	1.19 (0.81–1.74)	1.05 (0.70–1.58)
Serotypes in PCV7				
Non-carriers	107 (43.8)	137 (56.2)	1 (reference)	1 (reference)
Carriers	88 (49.2)	91 (50.8)	1.24 (0.84–1.82)	1.08 (0.72–1.62)
Six additional serotypes in PCV13				
Non-carriers	162 (46.7)	185 (53.3)	1 (reference)	1 (reference)
Carriers	33 (43.4)	43 (56.6)	0.88 (0.53–1.45)	0.76 (0.45–1.28)
Subgroup aged <10 years	(*n* = 95)	(*n* = 81)		
Any serotype				
Non-carriers	45 (54.2)	38 (45.8)	1 (reference)	1 (reference)
Carriers	50 (53.8)	43 (46.2)	0.98 (0.54–1.78)	1.01 (0.55–1.86)
Serotypes in PCV7				
Non-carriers	49 (55.1)	40 (44.9)	1 (reference)	1 (reference)
Carriers	46 (52.9)	41 (47.1)	0.92 (0.51–1.66)	0.93 (0.51–1.72)
Six additional serotypes in PCV13				
Non-carriers	74 (54.4)	62 (45.6)	1 (reference)	1 (reference)
Carriers	21 (52.5)	19 (47.5)	0.93 (0.46–1.88)	0.98 (0.47–2.03)
Subgroup aged 10–14 years	(*n* = 87)	(*n* = 120)		
Any serotype				
Non-carriers	47 (40.2)	70 (59.8)	1 (reference)	1 (reference)
Carriers	40 (44.4)	50 (55.6)	1.19 (0.68–2.08)	1.08 (0.61–1.94)
Serotypes in PCV7				
Non-carriers	47 (38.8)	74 (61.2)	1 (reference)	1 (reference)
Carriers	40 (46.5)	46 (53.5)	1.37 (0.78–2.40)	1.22 (0.68–2.20)
Six additional serotypes in PCV13				
Non-carriers	76 (43.7)	98 (56.3)	1 (reference)	1 (reference)
Carriers	11 (33.3)	22 (66.7)	0.65 (0.29–1.41)	0.54 (0.23–1.26)

*95* % *CI* 95 % confidence interval; *OR* odds ratio; *PCV7* 7-valent pneumococcal conjugate vaccine; *PCV13* 13-valent pneumococcal conjugate vaccine

^a^ORs adjusted for age (as a continuous term), gender, ethnicity, number of siblings, and parental smoking

Table 4 shows the individual serotypes identified by vaccination status. Only 10 children (four previously vaccinated with PCV7 and six unvaccinated) were exclusively colonised by serotypes not included in PCV13. Among the vaccinated children colonised by *S. pneumoniae*, respectively 34.4, 44.1, and 17.2 % were carriers of one, two or ≥3 PCV13 serotypes; the corresponding figures among the unvaccinated patients were 29.2, 28.2 and 36.6 %. The use of PCV7 did not influence carriage of one or two PCV13 serotypes, but the proportion of PCV7-vaccinated children colonised with ≥3 PCV13 serotypes was significantly lower than that of unvaccinated children (OR 0.46; 95 % CI 0.23–0.91).

**Table 4:** Carriage of specific pneumococcal subtypes in children with asthma, by pneumococcal vaccination status

	Vaccinated with PCV7, n (%)	Not vaccinated with PCV7, n (%)	Crude OR (95 % CI)	OR (95 % CI)^a^
Total carriers	93	99		
Carriers of serotypes not included in PCV13	4 (4.3)	6 (6.0)	0.84 (0.23–3.07)	0.97 (0.25–3.77)
Carriers of PCV13 serotypes				
1	32 (34.4)	29 (29.2)	1.40 (0.80–2.47)	1.23 (0.69–2.21)
2	41 (44.1)	28 (28.2)	1.86 (1.08–3.21)	1.64 (0.93–2.89)
≥3	16 (17.2)	36 (36.6)	0.57 (0.30–1.08)	0.46 (0.23–0.91)
Carriers of different PCV13 serotypes				
Serotype 1	1 (1.0)	2 (2.0)	0.58 (0.05–6.47)	0.56 (0.05–6.40)
Serotype 3	12 (13.0)	11 (11.1)	1.29 (0.56–3.00)	1.18 (0.50–2.80)
Serotype 4	26 (28.0)	39 (39.4)	0.75 (0.43–1.28)	0.62 (0.36–1.09)
Serotype 5	15 (16.1)	23 (23.2)	0.74 (0.38–1.47)	0.68 (0.33–1.38)
Serotype 6A	5 (5.4)	6 (6.1)	0.97 (0.29–3.24)	0.65 (0.18–2.40)
Serotype 6B	0 (0.0)	3 (3.0)	NE	NE
Serotype 7 F	2 (2.1)	4(4.0)	0.58 (0.10–3.20)	0.60 (0.10–3.49)
Serotype 9 V	18 (19.3)	22 (22.1)	0.95 (0.49–1.83)	0.95 (0.48–1.87)
Serotype 14	0 (0.0)	1 (1.0)	NE	NE
Serotype 18C	0 (0.0)	0 (0.0)	NE	NE
Serotype 19A	3 (3.2)	7 (7.1)	0.49 (0.13–1.94)	0.43 (0.11–1.77)
Serotype 19 F	83 (89.2)	84 (84.9)	1.27 (0.86–1.88)	1.13 (0.75–1.70)
Serotype 23 F	2 (2.1)	2 (2.0)	1.17 (0.16–8.40)	0.83 (0.11–6.26)

Reference category is non-carrier of corresponding serotypes

*95* % *CI* 95 % confidence interval; *NE* not estimable; *OR* odds ratio; *PCV7* 7-valent pneumococcal conjugate vaccine; *PCV13* 13-valent pneumococcal conjugate vaccine

^a^ORs adjusted for age (as a continuous term), gender, ethnicity, number of sibling, and parental smoking

Serotypes 19 F, 4 and 9 V were the most frequently identified serotypes in the vaccinated patients, and these and serotype 5 were most frequently detected in the unvaccinated patients. None of the serotypes included in PCV7 or PCV13 was associated with vaccination status.

## Discussion

The findings of this study show that a considerable number of school-aged children and adolescents with asthma are colonised by *S. pneumoniae*. The highest colonization rates were found among males and this is not surprising because a strict association between pneumococcal carriage and male gender has been already reported by Cardozo et al. in healthy subjects of the same age [13]. The global high rates of detection may be explained by the fact that we used the most accurate available methods. Respiratory secretions were collected from the oropharynx which has been demonstrated to be the most effective site for detecting *S. pneumoniae* in older children and adults [14–16]. In a recent study, it was shown that oropharyngeal sampling appeared significantly more effective than nasopharyngeal sampling in identifying and characterizing pneumococcal carrier status in adolescents [14]. Moreover, a flocked nylon fibre tip was used because previous studies have shown that this ensures the highest rate of detection of *S. pneumoniae*, particularly in comparison with the more widely used Dacron and rayon swabs) [16]. *S. pneumoniae* was identified by means of molecular methods that, albeit with some exceptions [17], have been found to be significantly more reliable than traditional non-enriched cultures in routine practice [18]. Furthermore, to improve the detection of *S. pneumoniae* without increasing the risk of false-positive results, both the *lytA* and *cpsA* genes were amplified [19, 20]. This permitted to avoid false-positive results because only *S. pneumoniae* has both these genes, whereas different streptococci that are not capsulated but that could colonies oropharynx possess only lytA gene but not cpsA gene [19, 20].

It has been previously shown that pharyngeal bacterial colonisation is associated with an increased risk of asthma. Bisgaard et al., who cultured pharyngeal samples taken from 321 healthy 1-year-olds followed up for 5 years found that those colonised by *S. pneumoniae* and/or other bacterial respiratory pathogens such as *Haemophilus influenzae* and *Moraxella catarrhalis* were significantly more prone to develop persistent wheezing (hazard ratio [HR] 2.40; 95 % CI 1.45–3.99), to experience severe wheezing exacerbations (HR 2.99; 95 % CI 1.66–5.33), and to be hospitalised because of wheezing (HR 3.85; 95 % CI 1.90–7.99) [21]. Moreover, children with asthma seem to have a primary immune response defect that favours pneumococcal colonization. Larsen et al. took peripheral blood mononuclear cells (PBMCs) from infants aged 6 months, and stored them until the children were 7 years old [22]; at this time, after stimulation with *Haemophilus influenzae*, *Moraxella catharralis* and *S. pneumoniae*, the PBMCs taken from the future asthmatic subjects showed the aberrant production of interleukin(IL)-5 (*p* = 0.008), IL-13 (*p* = 0.057), IL-17 (*p* = 0.001), and IL-10 (*p* = 0.028). The hypothesis that colonization is favoured by a reduction in immune system efficiency of subjects that later develop asthma is further supported by the findings of Jung et al. [23], who evaluated serotype-specific pneumococcal antibody levels after the administration of PPSV23 and found that the immune response to pneumococcal antigens was defective in asthmatic young adults in comparison with healthy subjects. Furthermore, subjects with atopic conditions other than asthma (i.e. atopic dermatitis, allergic rhinitis, and hay fever) also have an impaired immune response to *S. pneumoniae* [24]. Their underlying immunological mechanisms are similar to those of asthmatic children (i.e., a Th2-predominant immune milieu) and they are significantly less able to produce specific antibody responses to PPSV23 [25], and they are at increased risk of severe pneumococcal disease [26].

As expected, the prevalence of pneumococcal colonisation in our asthmatic subjects was strictly age-related and was significantly lower in those who had recently received antibiotic therapy. A progressive reduction in pneumococcal colonisation with increasing age has been repeatedly found in healthy children [27], including school-aged children and adolescents [28], and can be explained by the maturation of the immune system and the progressive reduction of the role of factors such as day-care attendance that can significantly enhance the horizontal spread of pneumococcal strains.

Finally, carriage did not correlate with the severity of asthma or variables such as the use of systemic corticosteroids or respiratory relapses in the period preceding pharyngeal sampling (which were more frequent among the patients with the severest asthma). If carriage is *per se* a prerequisite for IPD, this seems to suggest that all asthmatic children are at risk and that, although not officially recommended [3], all of them should undergo pneumococcal vaccination, not just the severe cases receiving long-term corticosteroid therapy. However, further studies are needed with a large sample size of children with severe asthma in order to identify factors that influence pneumococcal colonization in these patients.

We did not find any statistically significant difference in the carriage of particular pneumococcal serotypes between the children previously vaccinated or not with PCV7 in the population as a whole or in the subgroups aged <10 or 10–14 years. Although only one sample was taken from each child and we did not evaluate pneumococcal colonisation over time, this seems to indicate that the efficacy of PCV7 against colonisation does not persist among children immunised in the first years of life, at least not when vaccine uptake is relatively low and more than 40 % of classmates remain unvaccinated. This finding, that confirms what has been recently found in healthy school-aged children and adolescents [28], is different from that reported by Le Polain de Waroux et al. [29] and it is not easy to explain. Le Polain de Waroux et al., who studied nasopharyngeal carriage in children vaccinated with PCV7, found that this vaccine conferred reasonable protection against acquisition of pneumococcal carriage of the 7 serotypes included in the vaccine for several years. However, they studied children who have received the vaccine 5–64 months after the last PCV7 dose, a period shorter than that considered in this study. It can be hypothesised that the antibody levels evoked in the first years of life progressively decrease over time and after several years, as in this study, become no longer adequate to avoid colonisation. On the contrary, no role at this regard had probably the lower number of PCV7 doses received by the children enrolled in this study. Despite it was reported that 3 primary doses as in the study by La Polain de Waroux et al. [29] could reduce carriage more than 2 primary doses as in this study, the differences were found borderline and statistically significant only in one of the three comparative studies that have evaluated the duration of protection against carriage according to the number of PCV7 doses received by the children [30]. Because each serotype has different putative correlates of protection [31], it is likely that those which need the highest concentrations are those more frequently carried. We found that the most commonly detected serotype was 19 F, which has been reported to have a very high correlate of protection against IPD [32]. On the other hand, Simell et al. [31] studied the associations between pneumococcal nasopharyngeal carriage and the serum concentrations of serotype-specific antibodies in toddlers 1 month after they had been immunised with a 9-valent PCV, and found that higher anti-serotype 14 and 19 F IgG and anti-serotype 14 IgM correlated with a lower probability of pneumococcal acquisition. However, the real relevance of the pharyngeal re-colonization by the same serotypes included in the previously used vaccine in conditioning risk of disease in vaccinated children and their households has to be established. Further studies at this regard are needed.

About 95 % of the children colonised by *S. pneumoniae* carried at least one of the serotypes included in PCV13, and only 10 cases were colonised only with non-typeable serotypes. This seems to indicate that PCV13 might be to be potentially efficacious in preventing IPD in children with asthma and booster doses have to be administered in order to avoid the risk or recolonization. However, further studies are needed to evaluate whether it can be administered alone or in combination with PPV23 and whether and when booster doses have to be periodically administered.

A limitation of this study is represented by the absence of a control group of healthy children and this does not permit to evaluate whether children with asthma were differently colonized in comparison to normal subjects living in the same geographic area with limited pneumococcal vaccination coverage.

## Conclusions

The carriage of *S. pneumoniae* is common in all school-aged children and adolescents with asthma, regardless of the severity of the disease and the administration of PCV7 in the first years of life. This highlights the problem of the duration of the protection against colonisation provided by pneumococcal conjugate vaccine, and the role of pharyngeal recolonization from a clinical point of view.

## Abbreviations

95 % CI: 95 % confidence intervals
HR: hazard ratio
IL: interleukin
IPD: invasive pneumococcal disease
OR: odds ratio
PBMC: peripheral blood mononuclear cells
PCR: polymerase chain reaction
PCV7: 7-valent pneumococcal conjugate vaccine
PCV13: 13-valent pneumococcal conjugate vaccine
PPSV23: 23-valent polysaccharide vaccine (PPSV23)
